# Two inequalities about the pedal triangle

**DOI:** 10.1186/s13660-018-1661-7

**Published:** 2018-04-03

**Authors:** Fangjian Huang

**Affiliations:** School of Automation Engineering, University of Electronic Science and Technology of China, Chengdu, P.R. China

**Keywords:** 51M16, 12-04, Interior point, Pedal triangle, Inequality, Automated inequality proving

## Abstract

Two conjectures about the pedal triangle are proved. For the first conjecture, the product of the distances from an interior point to the vertices is mainly considered and a lower bound is obtained by the geometric method. To prove the other one, an analytic expression of the distance between the circumcenter and an interior point is achieved by the distance geometry method. A procedure to transform the geometric inequality to an algebraic one is presented. And then the proof is finished with the help of a Maple package, Bottema. The proof process could be applied to similar problems.

## Introduction

**Figure 1:**
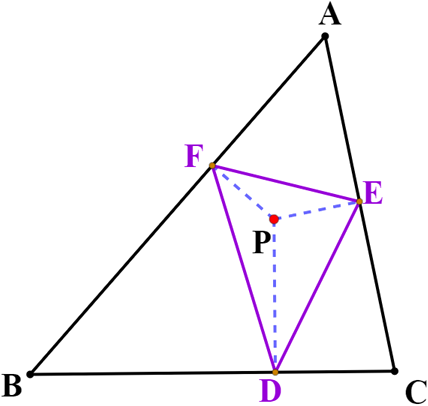
Pedal triangle. The pedal triangle of the interior point *P* with respect to triangle $ABC$

For an interior point *P* of a triangle $ABC$, let *D*, *E*, *F* denote the feet of the perpendiculars from *P* to *BC*, *CA*, *AB* (may be produced), respectively. Then the triangle $DEF$ is the pedal triangle of *P* with respect to $\triangle ABC$ shown in Fig. 1. It is an elementary geometric object and has been introduced in many textbooks such as [1] and [2], in which lots of theorems about the pedal triangle were presented. Most of these results are equalities. In [3], Liu puts forward some inequalities involving pedal triangles.

**Figure 2:**
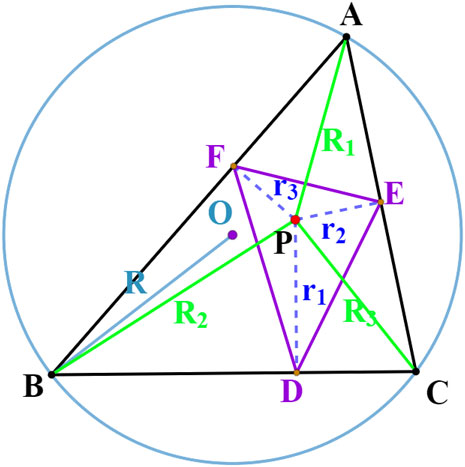
Notations. Notations of an interior point of a triangle

Let *O*, *R*, *r*, *S* denote the circumcenter, circumradius, inradius, and the area of $\triangle ABC$, respectively; *a*, *b*, *c* denote the lengths of line segments *BC*, *CA*, *AB*; $R_{1}$, $R_{2}$, $R_{3}$, $r_{1}$, $r_{2}$, $r_{3}$ denote the distances from *P* to *A*, *B*, *C*, *D*, *E*, *F*, respectively; and $R_{p}$ denotes the circumradius of $\triangle DEF$, shown in Fig. 2.

In the last section of [3], some conjectures were presented. For conjectures (3.4) and (3.5), we determine that they are both correct. Notations as above, these two conjectures are as follows:

1 $$ PO\geq|R-2\cdot R_{p}| $$

and

2 $$ \frac{R_{1}R_{2}R_{3}}{r_{1}r_{2}r_{3}}\geq\frac{8\cdot R_{p}^{2}}{r^{2}}. $$

Here, *PO* is the distance from *P* to the circumcenter *O*. (Actually, $|PO|$ is more formal, however, we usually use *PO* when there is no confusion.)

Although these two conjectures are about $R_{p}$, the circumradius of $\triangle DEF$, by the following well-known equation (Theorem 198, Corollary C, [2]),

3 $$ R_{p}=\frac{R_{1}R_{2}R_{3}}{2\cdot(R^{2}-PO^{2})}, $$

we get an equivalent inequality of (1)

4 $$ (R-PO) (R+PO)^{2}\geq R_{1}R_{2}R_{3} \geq(R-PO)^{2}(R+PO). $$

There are only three geometric variables involved above. One is $R_{1}R_{2}R_{3}$, the product of the distances from *P* to *A*, *B*, *C*, the other two are the circumradius of $\triangle ABC$ and the distance from *P* to *O*. We could prove it in a geometric way.

For conjecture (2), an equivalent inequality by (3) is

5 $$ R_{1}R_{2}R_{3}\leq \frac{r^{2}\cdot(R^{2}-PO^{2})^{2}}{2\cdot r_{1}r_{2}r_{3}}. $$

However, there are more variables in this inequality. We prove it in an algebraic way instead of a geometric one.

The remaining parts are arranged as follows. First, according to the position of circumcenter, we prove conjecture (4) in three subcases in Sect. 2. After that, an analytic expression of *PO* is obtained by the distance geometry method [4]. Based on this expression, conjecture (2) is transformed and proved with the help of a Maple package Bottema [5] in Sect. 3. We also compare these two upper bounds of $R_{1}R_{2}R_{3}$ in the last part.

## Proof to the first conjecture

In this section, we present a geometric proof to conjecture (1). First, we recall a result, Theorem 2 of [6] for the left-hand side inequality of (4). For the right-hand side, we divide it into three subcases to construct this lower bound of $R_{1}R_{2}R_{3}$ according to the position of *O* in Proposition 1.

### An upper bound of $R_{1}R_{2}R_{3}$

For a point in a polytope, [6] presented an upper bound of the product of the distances from the vertices. We just list them here.

#### Lemma 1

(Theorem 2 in [6])

*Let* $\boldsymbol {x},\boldsymbol {x}_{1},\ldots,\boldsymbol {x}_{m}$ ($m\geq2$) *be* (*not necessarily distinct*) *points of the solid unit sphere* $\boldsymbol {U}_{n}$ *of* $\boldsymbol {E}_{n}$ *such that* ***x*** *belongs to the convex hull of* $\boldsymbol {x}_{1},\ldots,\boldsymbol {x}_{m}$. *Then*

6 $$ \prod_{i=1}^{m}\|\boldsymbol {x}-\boldsymbol {x}_{i}\|\leq\bigl(1-\|\boldsymbol {x}\|\bigr)\cdot\bigl(1+\|\boldsymbol {x}\|\bigr)^{m-1}. $$

*For* $0<\|\boldsymbol {x}\|<1$, *equality holds in* (6) *only under the following conditions*: $\|\boldsymbol {x}_{i}\|=1$, $i=1,\ldots,m$, $m-2$ *of* $\boldsymbol {x}_{i}$ *coincide with the point* $a(\boldsymbol {x})$ *of* $\boldsymbol {U}_{n}$ *farthest away from* ***x***, *and* ***x*** *lies on the chord bounded by the two remaining points*.

In this lemma, $\boldsymbol {E}_{n}$ denotes the real *n*-dimensional Euclidean space and $\|\boldsymbol {x}\|$ is the Euclidean norm of ***x***.

When considering a triangle $ABC$ and a point *P* that belongs to it, based on this lemma, we have

7 $$ R_{1}R_{2}R_{3}\leq(R-PO) \cdot(R+PO)^{2}, $$

and the equality holds if and only if one of the following conditions holds:

1. *P* lies on the chord joining two points of $\{A,B,C\}$, and the remaining one is farthest away from *P* on the circumcircle of $\triangle ABC$.
2. The circumcenter *O* is inside $\triangle ABC$ and *P* coincides with *O*.
3. *P* coincides with one of the vertices of $\triangle ABC$.

That is to say, when *P* is an interior point of $\triangle ABC$, we have inequality (7) and the equality holds only when *P* coincides with *O*.

### A lower bound of $R_{1}R_{2}R_{3}$

For the right-hand side of (4), we have the following.

#### Proposition 1

*Notations as above*, *for any interior point* *P* *of* $\triangle ABC$, *we have*

8 $$ R_{1}R_{2}R_{3} \geq(R-PO)^{2}\cdot(R+PO), $$

*and the equality holds only when* *P* *coincides with the circumcenter* *O*.

#### Proof

We discuss this problem in three cases according to the position of *O*.

*I.* *O* is outside $\triangle ABC$.

**Figure 3:**
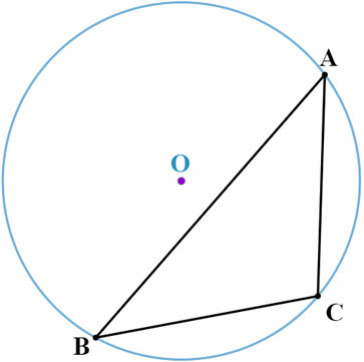
*O* is outside. The circumcenter is outside the triangle

In this case, there must exist one side of $\triangle ABC$ (e.g., *AB*) such that *O* and the remaining point (*C*) are located on its different sides (see Fig. 3).

**Figure 4:**
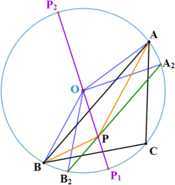
Auxiliary lines. Auxiliary lines when *O* is outside $\triangle ABC$

For any interior point *P* of $\triangle ABC$, draw a line passing through *P*, parallel to *AB* and meeting the circumcircle in two points, then one point must be on the minor arc $\overset{⏜}{AC}$ and the other must be on the minor arc $\overset{⏜}{CB}$. Let $A_{2}$ denote the first one and the latter is denoted by $B_{2}$. Produce *OP* to intersect the circumcircle at two points $P_{1}$ and $P_{2}$ in which $P_{1}$ is on the minor arc $\overset{⏜}{ACB}$ and $P_{2}$ is on the major arc $\overset{⏜}{AB}$. Details are shown in Fig. 4. Then we have:

- $P_{1}P_{2}$ is a diameter of the circumcircle.
- $P_{1}$ is on the minor arc $\overset{⏜}{B_{2}CA_{2}}$.
- The minor arc $\overset{⏜}{P_{1}A_{2}}$ is smaller than the arc $\overset{⏜}{P_{1}A_{2}A}$.
- The minor arc $\overset{⏜}{P_{1}B_{2}}$ is smaller than the arc $\overset{⏜}{P_{1}B_{2}B}$.

Therefore, $\angle POA_{2}=\angle P_{1}OA_{2}<\angle P_{1}OA=\angle POA$ and $\angle POB_{2}=\angle P_{1}OB_{2}<\angle P_{1}OB=\angle POB$. Let us compare $\triangle POA_{2}$ and $\triangle POA$. There is a common side *PO*. $OA_{2}$ and *OA* are both *R*, the circumradius. According to the law of cosines, we have $PA>PA_{2}$. Similarly, we have $PB>PB_{2}$. Since the chord $A_{2} B_{2}$ and the diameter $P_{2} P_{1}$ intersect at the point *P*, according to the intersecting chords theorem (also known as power of a point or secant tangent theorem), we have $PA_{2}\cdot PB_{2}=PP_{1}\cdot PP_{2}=(R-PO)\cdot(R+PO)$. Additionally, we have $PC\geq|OC-OP|=R-PO$ and the equality holds only when *P* lies on the radius *OC*. Then there exist

9 $$ R_{1} R_{2} R_{3}=PA\cdot PB\cdot PC>PA_{2}\cdot PB_{2}\cdot PC\geq(R-PO)^{2} \cdot(R+PO). $$

*II.* *O* is on a side of $\triangle ABC$.

Assume that *O* is on the side *AB* of $\triangle ABC$. Draw a line passing through *P* and parallel to *AB*. By a similar way, we can prove (8) for any interior point *P* of $\triangle ABC$.

*III.* *O* is inside $\triangle ABC$.

In this case, we need partition $\triangle ABC$ to three quadrilaterals. Produce *AO*, *BO*, *CO* to meet *BC*, *CA*, *AB* in $A_{1}$, $B_{1}$, $C_{1}$ respectively, so *P* must lie inside one of the quadrilaterals $CB_{1}OA_{1}C$, $B_{1} AC_{1} OB_{1}$ and $C_{1} BA_{1} OC_{1}$, or on $OA_{1}$ or $OB_{1}$ or $OC_{1}$.

When *P* coincides with *O*, $PO=0$ and $R_{1}=R_{2}=R_{3}=R$, then the equality of (8) holds.

**Figure 5:**
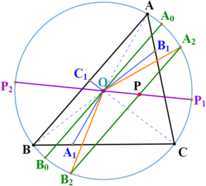
*O* is inside. Auxiliary lines when *P* is in the quadrilateral $CB_{1}OA_{1}C$

When *P* lies in a quadrilateral, say $CB_{1} OA_{1}C$ (see Fig. 5), let us draw a line which is parallel to *AB*, passes through *P* and meets the circumcircle in two points in which one is on the minor arc $\overset{⏜}{CA}$ and the other is on the minor arc $\overset{⏜}{BC}$. Let $A_{2}$ and $B_{2}$ denote the former and the latter, respectively. Draw a line from *O* to *P* and produce it to intersect the circumcircle at $P_{1}$. Draw another line from *P* to *O* and produce it to meet the circumcircle again in $P_{2}$. And draw a line passing through *O*, parallel to *AB* and meeting the circumcircle in two points. Let $A_{0}$ ($B_{0}$) denote the point on the minor arc $\overset{⏜}{CA}$ ($\overset{⏜}{BC}$). Therefore, the following properties are easy to prove:

- $A_{2}$ lies on the minor arc $\overset{⏜}{CA_{0}}$ and $B_{2}$ lies on the minor arc $\overset{⏜}{B_{0}C}$.
- $P_{2}$ lies on the minor arc $\overset{⏜}{AB}$ and $P_{1} P_{2}$ is a diameter of the circumcircle.
- $P_{1}$ lies on the minor arc $\overset{⏜}{B_{2}CA_{2}}$.
- $\angle P_{1} OA_{2}<\angle P_{1} OA_{0}<\angle P_{1} OA$ and $\angle P_{1} OB_{2}<\angle P_{1} OB_{0}<\angle P_{1} OB$.

Once again, comparing $\triangle POA_{2}$ and $\triangle POA$, there is a common side *PO* and *OA*, $OA_{2}$ are both circumradius, then we have $PA>PA_{2}$ according to the law of cosines. Similarly, we could have $PB>PB_{2}$. Applying the intersecting chords theorem to the chord $A_{2} B_{2}$ and the diameter $P_{1} P_{2}$, we have

10 $$ PB_{2}\cdot PA_{2} =PP_{1}\cdot PP_{2}=(R-PO) (R+PO). $$

Additionally, $PC\geq OC-PO=R-PO$ and the equality holds only when *P* lies on the radius *OC* of the circumcircle, we have

11 $$ R_{1} R_{2} R_{3}=PA\cdot PB\cdot PC>PA_{2}\cdot PB_{2}\cdot PC\geq(R-PO)^{2} (R+PO). $$

When *P* is in the quadrilateral $B_{1} AC_{1} OB_{1}$ ($C_{1} BA_{1} OC_{1}$), we draw the parallel line of *BC* (*AC*) through *P*. When *P* is on the line segment $OA_{1}$ ($OB_{1}$, $OC_{1}$) and does not coincide with *O*, we draw the parallel line of *AB* (*BC*, *AC*) through *P*, respectively. And in a similar way, we could obtain (8).

From all above, we achieve that (8) holds for every interior point *P* of $\triangle ABC$ and the equality holds only when *P* coincides with *O*. □

Based on (7), (8), and (4), we determine that (1) is correct for any interior point *P* of $\triangle ABC$ and the equality takes place if and only if *P* coincides with the circumcenter.

## Proof to the second conjecture

First we use the barycentric coordinate system and the distance geometry method to present an analytic expression of *PO*. And then we transform conjecture (2) equivalently to a polynomial inequality with four variables. After that, an inequality proving tool, Maple package Bottema developed by Prof. Lu Yang and his collaborators, is invoked to help us prove it.

Let $(x,y,z)$ denote the barycentric coordinates of the interior point *P* with respect to $\triangle ABC$. And we choose the normalized coordinates. That is to say,

12 $$ x=\frac{S_{\triangle PBC}}{S},\qquad y=\frac{S_{\triangle PCA}}{S},\qquad z=\frac {S_{\triangle PAB}}{S}, $$

in which $S_{\triangle PBC}$ denotes the area of the triangle $PBC$, similar as $S_{\triangle PCA}$ and $S_{\triangle PAB}$ do. Therefore, we have $x+y+z=1$. There are also some well-known formulas, we just list them below without proof.

13 $$\begin{aligned}& S=\frac{1}{4}\sqrt{(a+b+c) (b+c-a) (a+c-b) (a+b-c)}, \end{aligned}$$

14 $$\begin{aligned}& r=\frac{2 S}{a+b+c},\qquad R=\frac{abc}{4S},\qquad r_{1}=\frac{2S\cdot x}{a},\qquad r_{2}=\frac{2S\cdot y}{b},\qquad r_{3}=\frac{2S\cdot z}{c}, \end{aligned}$$

15 $$\begin{aligned}& R_{1}=\sqrt{b^{2}z^{2}+c^{2}y^{2}+yz \bigl(b^{2}+c^{2}-a^{2}\bigr)}, \end{aligned}$$

16 $$\begin{aligned}& R_{2}=\sqrt{c^{2}x^{2}+a^{2}z^{2}+xz \bigl(a^{2}+c^{2}-b^{2}\bigr)}, \end{aligned}$$

17 $$\begin{aligned}& R_{3}=\sqrt{a^{2}y^{2}+b^{2}x^{2}+xy \bigl(a^{2}+b^{2}-c^{2}\bigr)}. \end{aligned}$$

What we need more is an explicit expression of *PO*.

### Lemma 2

*For any interior point* *P* *of* $\triangle ABC$, *notations as above*, *we have*

18 $$ PO=\sqrt{\frac {a^{2}R_{1}^{2}(b^{2}+c^{2}-a^{2})+b^{2}R_{2}^{2}(a^{2}+c^{2}-b^{2})+c^{2}R_{3}^{2}(a^{2}+b^{2}-c^{2})-a^{2}b^{2}c^{2} }{(a+b+c)(b+c-a)(a+c-b)(a+b-c)}}. $$

### Proof

We use the distance geometry method to achieve this equation.

Let $\triangle ABC$ be the reference triangle, for any point *Q* on the plane of $\triangle ABC$, we take

19 $$ \bigl(QA^{2},QB^{2},QC^{2}\bigr) $$

as the coordinates of *Q* w.r.t. $\triangle ABC$. Here, $QA^{2}$ ($QB^{2}$, $QC^{2}$) is the square of the distance between *Q* and *A* (*B*, *C*). Then the Cayley–Menger matrix of *A*, *B*, *C*, *O*, *P* is

20 $$ \mathit{CM}=\left ( \textstyle\begin{array}{c@{\quad}c@{\quad}c@{\quad}c@{\quad}c@{\quad}c} 0&c^{2}&b^{2}&R^{2}&R_{1}^{2}&1\\ c^{2}&0&a^{2}&R^{2}&R_{2}^{2}&1\\ b^{2}&a^{2}&0&R^{2}&R_{3}^{2}&1\\ R^{2}&R^{2}&R^{2}&0&PO^{2}&1\\ R_{1}^{2}&R_{2}^{2}&R_{3}^{2}&PO^{2}&0&1\\ 1&1&1&1&1&0 \end{array}\displaystyle \right ). $$

Since these five points are on the same 2-dimensional plane, the $(4,5)$ minor of *CM* vanishes [7], i.e.,

21 $$ \left \vert \textstyle\begin{array}{c@{\quad}c@{\quad}c@{\quad}c@{\quad}c} 0&c^{2}&b^{2}&R^{2}&1\\ c^{2}&0&a^{2}&R^{2}&1\\ b^{2}&a^{2}&0&R^{2}&1\\ R_{1}^{2}&R_{2}^{2}&R_{3}^{2}&PO^{2}&1\\ 1&1&1&1&0 \end{array}\displaystyle \right \vert =0. $$

By solving this equation, we obtain

22 $$\begin{aligned}[b] PO^{2}={}&\frac{1}{(a+b+c)(b+c-a)(a+c-b)(a+b-c)}\cdot \bigl(a^{2}R_{1}^{2} \bigl(b^{2}+c^{2}-a^{2}\bigr) \\ &+b^{2}R_{2}^{2}\bigl(a^{2}+c^{2}-b^{2} \bigr)+c^{2}R_{3}^{2}\bigl(a^{2}+b^{2}-c^{2} \bigr)-2a^{2}b^{2}c^{2} \bigr)+R^{2}.\end{aligned} $$

Based on (13) and (14), we have

23 $$ R^{2}(a+b+c) (b+c-a) (a+c-b) (a+b-c)=a^{2}b^{2}c^{2}. $$

Consequently, we achieve (18). □

From (5), we also have another equivalent inequality of (2):

24 $$ r^{4}\cdot\bigl(R^{2}-PO^{2} \bigr)^{4}-4(r_{1} r_{2} r_{3}\cdot R_{1} R_{2} R_{3} )^{2}\geq0. $$

Substituting (13)–(18) into the left-hand side of (24), we get the following equivalent of (2):

25 $$ \frac{f}{16a^{2}b^{2}c^{2}\cdot(a+b+c)^{6}(b+c-a)^{2}(a+c-b)^{2}(a+b-c)^{2}}\geq0, $$

where

$$\begin{aligned} f= {}&\bigl( -x^{4}y^{4}z^{4}-x^{3}y^{5}z^{4}-x^{3}y^{4}z^{5}-x^{2}y^{5}z^{5} \bigr) a^{30}+ \bigl( -4x^{4}y^{4}z^{4} \\ &-4x^{3}y^{5}z^{4}-4x^{3}y^{4}z^{5}-4x^{2}{y}^{5}z^{5} \bigr) a^{29}b+ \bigl( -4x^{4}y^{4}z^{4}-4{x}^{3}y^{5}z^{4} \\ &-4x^{3}y^{4}z^{5}-4x^{2}y^{5}z^{5} \bigr) a^{29}c+ \bigl( -x^{5}y^{3}z^{4}+5x^{4}y^{4}z^{4}+6x^{3}y^{5}z^{4} \\ &+5x^{3}y^{4}z^{5}+x^{3}y^{3}{z}^{6}+5x^{2}y^{5}z^{5}+x^{2}y^{4}z^{6} \bigr) a^{28}b^{2}+ \bigl( -12x^{4}y^{4}z^{4} \\ &-12x^{3}y^{5}z^{4}-12x^{3}y^{4}z^{5}-12x^{2}y^{5}z^{5} \bigr) a^{28}bc+ \bigl( -x^{5}y^{4}z^{3}+5x^{4}y^{4}z^{4} \\ &+x^{3}y^{6}z^{3}+5x^{3}y^{5}z^{4}+6x^{3}y^{4}z^{5}+x^{2}y^{6}z^{4}+5x^{2}y^{5}z^{5} \bigr) a^{28}c^{2} \\ &+\cdots \\ &+ \bigl( -4x^{6}y^{4}z^{2}-3x^{6}y^{3}z^{3}+x^{6}y^{2}z^{4}+10x^{5}y^{5}z^{2}+10x^{5}y^{4}z^{3} \\ &+x^{5}y^{3}z^{4}+x^{5}y^{2}z^{5}+5x^{4}y^{5}z^{3}+11x^{4}y^{4}z^{4}+6x^{4}y^{3}z^{5} \bigr) b^{4}c^{26} \\ &+ \bigl( 4x^{6}y^{4}z^{2}+4x^{6}y^{3}z^{3}+40x^{5}{y}^{5}z^{2}+40x^{5}y^{4}z^{3}+44x^{4}y^{5}z^{3} \\ &+40x^{4}y^{4}z^{4}-4x^{4}y^{3}z^{5} \bigr) b^{3}c^{27}+ \bigl( x^{6}y^{4}z^{2}+x^{6}y^{3}z^{3}+5x^{5}y^{5}z^{2} \\ &+5x^{5}y^{4}z^{3}+6x^{4}y^{5}z^{3}+5x^{4}{y}^{4}z^{4}-x^{4}y^{3}z^{5} \bigr) b^{2}c^{28} \\ &+ \bigl( -4x^{5}y^{5}z^{2}-4x^{5}y^{4}z^{3}-4x^{4}y^{5}z^{3}-4x^{4}y^{4}z^{4} \bigr) bc^{29} \\ &+ \bigl( -x^{5}y^{5}z^{2}-x^{5}y^{4}z^{3}-x^{4}y^{5}z^{3}-x^{4}y^{4}z^{4} \bigr) c^{30}. \end{aligned}$$

Here, *f* is a homogeneous polynomial of degree 30 with respect to $\{ a, b,c\}$ with 496 terms, while *x*, *y*, *z* are treated as parameters. It is impractical for many algorithms and methods to prove $f\geq0$ directly, while there are some other constraints about *a*, *b*, *c*, *x*, *y*, and *z*.

Since *a*, *b*, *c* are the lengths of the three sides of $\triangle ABC$, we could use three positive variables *u*, *v*, *w* to express them as

26 $$ a=u+v,\qquad b=u+w,\qquad c=v+w. $$

Additionally, we could assume $a\geq b\geq c$ without loss of generality, and set

27 $$ a=u+v=1,\qquad b=(1-v)+w. $$

Therefore, we have

28 $$ u\geq v \geq w> 0,\quad v\in \biggl(0,\frac{1}{2} \biggr] $$

and

29 $$ v=\frac{1}{2+s},\qquad w=\frac{1}{t+\frac{1}{v}}=\frac{1}{2+s+t}, $$

in which $s,t$ are both non-negative real numbers. Because *P* is an interior point of $\triangle ABC$, $x,y,z$ should be positive and all less than 1. Since $x+y+z=1$, we can set

30 $$ z=1-x-y,\qquad x=\frac{1}{1+p},\qquad y=\frac{1}{q+\frac{1}{1-x}}= \frac {p}{1+p+p\cdot q}, $$

where *p* and *q* are both positive real numbers. Substituting (27),(29), and (30) into *f*, we achieve an equivalent inequality of $f\geq0$:

31 $$ \sum_{i=11}^{22} \Biggl( s^{i}\cdot\sum_{i=0}^{22-i} \bigl(h_{i,j}\cdot t^{j} \bigr) \Biggr)+\sum _{i=0}^{10} \Biggl( s^{i}\cdot\sum _{j=0}^{12} \bigl(h_{i,j}\cdot t^{j} \bigr) \Biggr)\geq0, $$

in which all the coefficients, $h_{i,j}$, are polynomials of *p* and *q*. Since *s*, *t* are non-negative and *p*, *q* are positive, we can use the function xprove in the Maple package Bottema to prove the positive semidefiniteness of $h_{i,j}$. Calculation shows that all these polynomials are positive semidefinite except $h_{0,1}$ and $h_{1,0}$. Then we verify the positive semidefiniteness of $h_{2,0} s^{2}+h_{1,0}s+h_{0,0}/2$ and $h_{0,2} t^{2}+h_{0,1} t+h_{0,0}/2$ by xprove. Both of them are confirmed. That is to say, (31) holds.

Because all the terms $h_{i,j}\cdot s^{i}t^{j}$ except $\{h_{0,1}t, h_{1,0}s\}$ are nonnegative, the polynomials $h_{2,0} s^{2}+h_{1,0}s+h_{0,0}/2$ and $h_{0,2} t^{2}+h_{0,1} t+h_{0,0}/2$ are nonnegative, the equality of (31) holds if and only if these things are all zero. Since *p*, *q* are both positive, there exist

32 $$\begin{aligned}& \begin{aligned}h_{22,0}={}&4p^{4}q^{4}\bigl(p^{2}+pq+2p+1 \bigr)^{2}\bigl(p^{2}q(p-2)^{2}+p^{3}q^{2}+p^{4}+p^{2}q^{2}+4p^{3} \\ &+p^{2}q+6p^{2}+2pq+4p+1\bigr) \bigl(p^{4}q+p^{3}q^{2}+p^{4}+12p^{3}q+p^{2}q^{2} \\ &+4p^{3}+5p^{2}q+6p^{2}+2pq+4p+1\bigr)\\>{}&0,\end{aligned} \\& h_{0,12}=(1+p)^{2}(pq+p+1)^{2}\bigl(4p^{3}q+p^{2}q^{2}+6p^{2}q+p^{2}+2pq+2p+1 \bigr)^{4}>0, \\& \begin{aligned}h_{0,0}={}&16\mbox{,}777\mbox{,}216\cdot \bigl( ( 1+p ) ^{2}{p}^{10} {q}^{10}+2 ( 2p+5 ) ( 1+p ) ^{3}{p}^{9} {q}^{9} \\ &+ {p}^{2}{q}^{2} \bigl( {p}^{6}{q}^{6}+ ( 1+p ) ^{6} \bigr) \bigl( 6{p}^{6}+56{p}^{5}-34{p}^{4}+153{p}^{3}+161{p}^{2} \\ & +212p+45 \bigr) + {p}^{3}{q}^{3} ( 1+p ) \bigl( {p}^{4}{q}^{4}+ ( 1+p ) ^{4} \bigr) \bigl( 4{p}^{7}+52{p}^{6}+37{p}^{5} \\ &-172{p}^{4}+85{p}^{3}-11{p}^{2}+592p+120 \bigr) \\ &+ {p}^{4}{q}^{4} ( 1+p ) ^{2} \bigl( {p}^{8}+20{p}^{7}-83{p}^{6}-45{p}^{5}-233{p}^{4}-113{p}^{3}-427{p}^{2} \\ &+1064p+210 \bigr) \bigl( {p}^{2}{q}^{2}+ ( 1+p ) ^{2} \bigr) \\ &+{p}^{5} ( 1+p ) ^{3}{q}^{5} \bigl( 2{p}^{8}+32{p}^{7}-15{p}^{6}-60{p}^{5}-240{p}^{4}-210{p}^{3}-650{p}^{2} \\ &+1288p+252 \bigr)+2p ( 2p+5 ) ( 1+p ) ^{11}q+ ( 1+p ) ^{12} \bigr)\\={}&0\end{aligned} \\& \quad\Leftrightarrow\quad p=2\wedge q=\frac{3}{2}. \end{aligned}$$

Therefore, the equality of (31) holds if and only if $s=t=0\wedge p=2 \wedge q=3/2$, i.e., $a=b=c=1\wedge x=y=z=1/3$.

From all above, (31) is proved, so are (25) and (24). That is to say, (2) is correct for $\triangle ABC$ and its interior point *P*, and the equality of (2) holds if and only if $\triangle ABC$ is an equilateral triangle and *P* is its circumcenter.

### Remark 1

Since $h_{0,0}\geq0$ when *p*, *q* are both positive, $h_{0,0}|_{p=0}=1$, $h_{0,0}|_{q=0}=(p+1)^{12}$, there must exist $\partial h_{0,0}/\partial p=0 \wedge\partial h_{0,0}/\partial q=0$, if the equality $h_{0,0}=0$ holds. Then we could use the Maple function RealRootCounting to show that there is only one real solution for the semialgebraic system $\{h_{0,0}=0, \partial h_{0,0}/\partial p=0, \partial h_{0,0}/\partial q=0, p>0, q>0\}$. Because $h_{0,0}|_{p=2, q=3/2}=0$, it just presents a proof to the equivalent (32).

### Remark 2

The function xprove in the Maple package Bottema is based on the dimensional-decreasing algorithm ([5], Chap. 8) and the complete discrimination system for polynomials [8]. It is quite a powerful tool for automated inequality proving; however, due to the expansion of symbolic computation, when there are too many variables and the degree is too high, the calculation will not be very efficient. The direct proof by xprove to $f\geq0$ is not practical. We tried for more than six hours, but nothing returned. In our proof, it takes three minutes to transform and prove the inequalities in Maple 2016 on a laptop with Intel I5-3230 CPU and 8GB RAM. This package is available at http://faculty.uestc.edu.cn/huangfangjian/en/article/167349/content/2378.htm.

### Remark 3

Inequalities (4) and (5) could both be treated as the upper bounds of $R_{1}R_{2}R_{3}$, the product of the distances from an interior point to the vertices of a triangle. Actually, we once tried to find the larger one between them, however, examples show that the comparison result varies. For example,

1. when $a=10$, $b=2$, $c=9$, $x=1/10$, $y=1/10$, $z=4/5$, we have
  33 $$\begin{aligned}& R_{1}R_{2}R_{3}\approx15,\qquad \frac{r^{2}(R^{2}-PO^{2})^{2}}{2r_{1}r_{2}r_{3}} \approx130,\qquad (R-PO) (R+PO)^{2}\approx92, \end{aligned}$$
2. when $a=10$, $b=6$, $c=8$, $x=1/2$, $y=1/3$, $z=1/6$, we have
  34 $$\begin{aligned}& R_{1}R_{2}R_{3}\approx88,\qquad \frac{r^{2}(R^{2}-PO^{2})^{2}}{2r_{1}r_{2}r_{3}} \approx115, \qquad(R-PO) (R+PO)^{2}\approx142. \end{aligned}$$

## Conclusion

In this paper, we have proved two interesting conjectures about the pedal triangle of an interior point of a triangle and analyzed the conditions when the equalities hold. We present a geometric method to deal with the first one. For the second one, we use some algebraic equations to transform it to a polynomial inequality and divide it into some inequalities with fewer variables and lower degrees. And then a computer-aided tool is invoked to finish the proof. As we know, there are plenty of inequality proving algorithms and methods. Taking advantages of these tools, we could think about complex issues. The procedure of the latter proof could be applied to other similar problems.

## References

[1] Gallatly W. (1910). The Modern Geometry of the Triangle.

[2] Johnson R.A. (1960). Advanced Euclidean Geometry.

[3] Liu J. (2013). On inequality $R_{p}< R$ of the pedal triangle. Math. Inequal. Appl..

[4] Yang L., Mucherino A., Lavor C., Liberti L., Maculan N. (2013). Solving spatial constraints with generalized distance geometry. Distance Geometry.

[5] Xia B., Yang L. (2016). Automated Inequality Proving and Discovering.

[6] Schwarz B. (1965). On the product of the distances of a point from the vertices of a polytope. Isr. J. Math..

[7] Sippl M.J., Scheraga H.A. (1986). Cayley–Menger coordinates. Proc. Natl. Acad. Sci. USA.

[8] Yang L., Hou X., Zeng Z. (1996). A complete discrimination system for polynomials. Sci. China Ser. E.

